# Gastric Cancer After Helicobacter pylori Eradication for Nodular Gastritis in an Adolescent

**DOI:** 10.7759/cureus.29984

**Published:** 2022-10-06

**Authors:** Takuma Okmaura, Yugo Iwaya, Mai Iwaya, Tomonobu Koizumi, Tadanobu Nagaya

**Affiliations:** 1 Department of Gastroenterology, Shinshu University, Matsumoto, JPN; 2 Department of Advanced Therapeutic Endoscopy, Shinshu University, Matsumoto, JPN; 3 Department of Laboratory Medicine, Shinshu University Hospital, Matsumoto, JPN; 4 Department of Hematology and Medical Oncology, Shinshu University, Matsumoto, JPN

**Keywords:** nodular gastritis, eradication therapy, gastric cancer, adolescent, helicobacter pylori

## Abstract

A 16-year-old girl underwent esophagogastroduodenoscopy (EGD) after the detection of *Helicobacter pylori *(*H. pylori*) antibodies in her urine during a school health screening, which revealed nodular gastritis (NG). She was diagnosed as having *H. pylori* infection histologically and by biopsy culture specimens and soon commenced eradication therapy. Eight weeks later, eradication was confirmed by a urea breath test. At the age of 19, however, she was referred to our hospital with epigastralgia and lower back pain. EGD revealed ulcerative lesions with enlarged folds at the greater curvature of the gastric body. Biopsy specimens of the lesions revealed poorly differentiated adenocarcinoma and signet ring cell carcinoma. The cancer was classified as stage IV with pancreatic invasion. Although NG with pangastritis is considered a high-risk factor for diffuse-type gastric carcinoma, the course of NG after eradication remains unknown. Careful histological assessment before eradication by endoscopic biopsy and close follow-up after eradication are therefore recommended, even in young patients.

## Introduction

Nodular gastritis (NG) is defined as antral gastritis with endoscopic findings typically characterized by a miliary pattern resembling “goose flesh” [1]. The histological hallmarks of NG are prominent lymphoid follicles [2,3]. Currently recognized as a type of *Helicobacter pylori* (*H. pylori*)-associated gastritis, NG is also considered a high risk factor for diffuse-type gastric carcinoma of the gastric body [4]. Although gastric cancer associated with NG in the teens or twenties has been described [5,6], reports are scarce on young patients with gastric cancer developing after eradication, and its clinical course remains unclear.

We herein describe the outcome of an adolescent who suffered diffuse-type gastric cancer only three years after successful *H. pylori* eradication for NG.

## Case presentation

**Figure 1:**
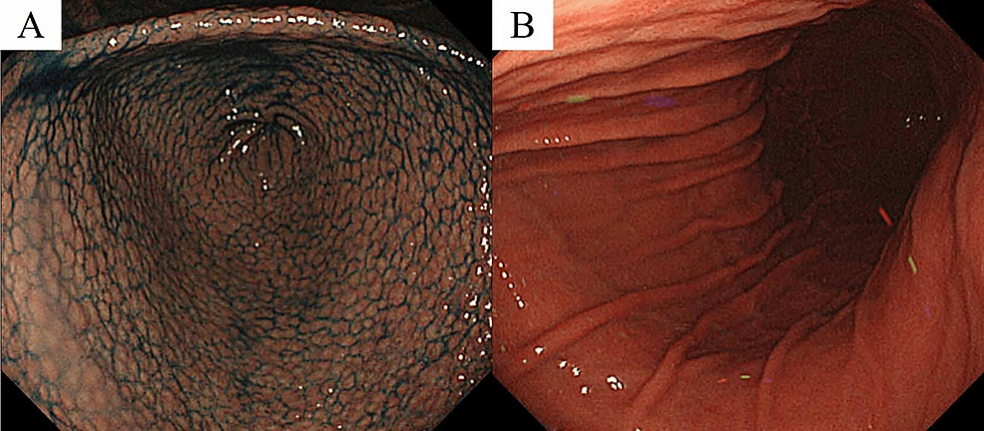
A) Initial esophagogastroduodenoscopy results revealed nodular gastritis in the antrum. B) No neoplastic changes were observed in the gastric body.

A 16-year-old girl was referred to our hospital after the detection of positivity for *H. pylori* antibodies in her urine during a school health screening. Although her medical history was unremarkable, her family history revealed *H. pylori *infection in her mother and gastric cancer in her grandmother. Esophagogastroduodenoscopy (EGD) disclosed NG and closed-type (C-2) atrophic gastritis according to the Kimura-Takemoto endoscopic classification system [7]. No neoplastic changes were detected (Figure 1).

**Figure 2:**
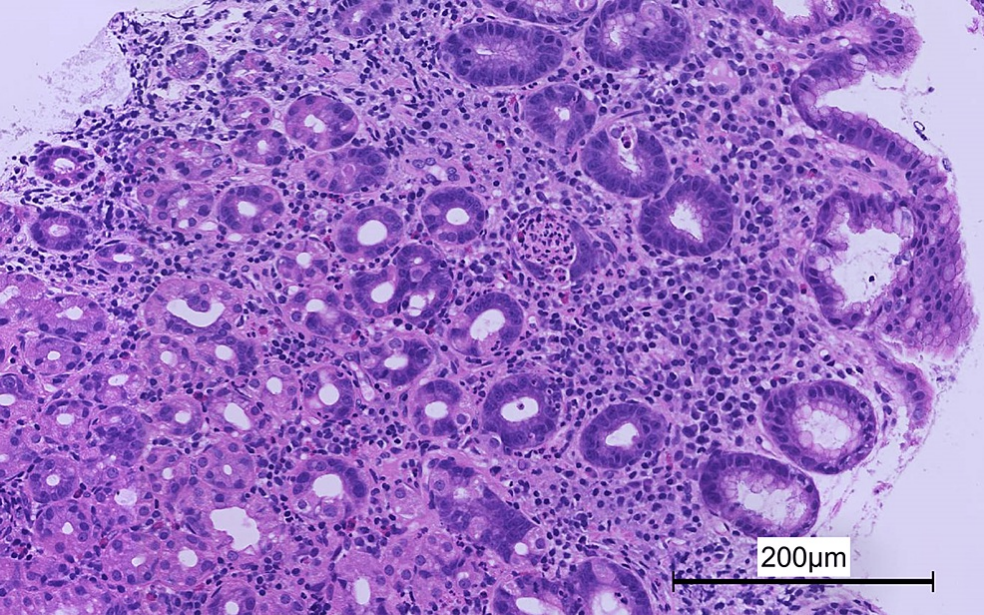
Low magnification (×100) of a biopsy specimen taken from the greater curvature of the gastric body. Moderate inflammatory cell infiltration was present (hematoxylin and eosin staining).

*H. pylori *infection was diagnosed based on histological findings and biopsy specimen culture. *H. pylori *genotype testing was not performed. Gastric biopsy samples taken from the gastric body revealed moderate inflammatory cell infiltration (Figure 2).

Since the *H. pylori* was resistant to clarithromycin in preliminary culture, we employed a second-line regimen consisting of a proton pump inhibitor, amoxicillin, and metronidazole. Eradication was confirmed by a 13C-urea breath test after eight weeks of therapy, and no further EGD was performed.

**Figure 3:**
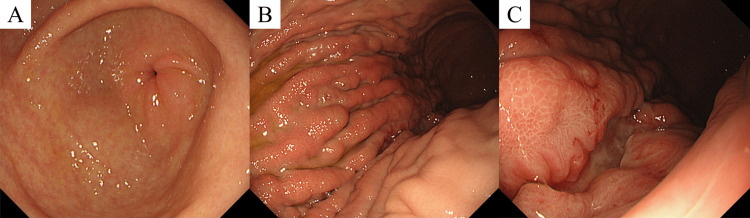
A) Esophagogastroduodenoscopy revealed improvement of the nodular gastritis. B) and C) Irregular ulcerative lesions with enlarged folds at the greater curvature of the gastric body were seen.

At the age of 19, she was referred to our hospital with epigastralgia and lower back pain. Although EGD confirmed the improvement of her NG, irregular ulcerative lesions with enlarged folds at the greater curvature of the gastric body were detected (Figure 3).

**Figure 4:**
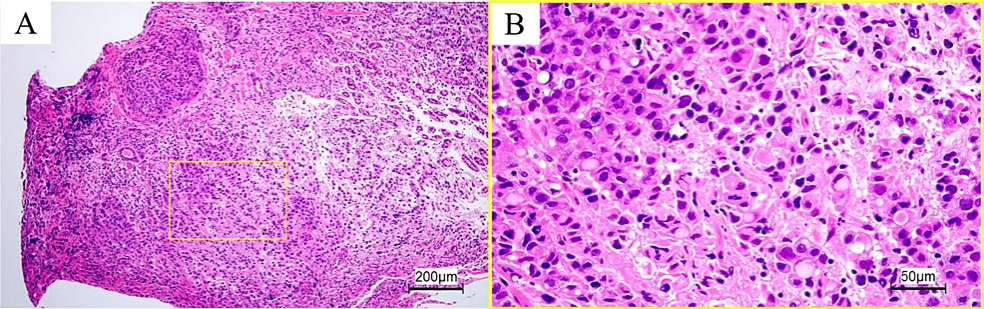
A: Low magnification (×100) of a biopsy specimen taken from an ulcerative lesion. B: Magnified image (×400) of the yellow square. Poorly differentiated adenocarcinoma and signet ring cell carcinoma were observed (hematoxylin and eosin staining).

Biopsy specimens of the lesions disclosed poorly differentiated adenocarcinoma and signet ring cell carcinoma (Figure 4). The patient’s human epidermal growth factor receptor 2 (HER2) was positive, although programmed death ligand 1, combined positive score, and microsatellite status were not tested. *H. pylori *was undetectable.

**Figure 5:**
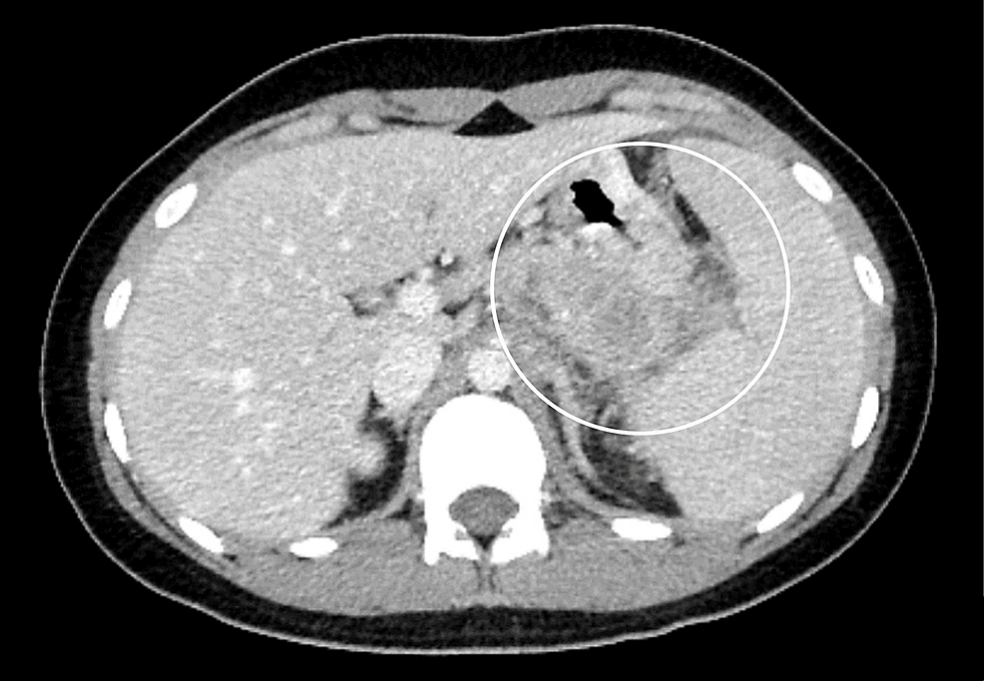
Abdominal CT revealed pancreatic and splenic arteriovenous infiltration (circle).

Enhanced abdominal CT revealed swollen intra-abdominal lymph nodes, pancreatic and splenic arteriovenous infiltration, and peritoneal dissemination (Figure 5).

Laboratory data showed no elevations of cancer embryonic antigen (CEA) or carbohydrate antigen (CA19-9). She was diagnosed as having stage IV cT4bN3M1 gastric adenocarcinoma according to the Japanese classification of gastric carcinoma [8]. In spite of systemic chemotherapy of trastuzumab combined with S-1 plus oxaliplatin as first-line treatment and nivolumab as second-line treatment, the disease worsened. She died eight months later.

## Discussion

We encountered a rare case of diffuse-type gastric cancer that developed in an adolescent only three years after successful *H. pylori *eradication for NG.

NG is currently thought to be an early-phase finding of* H. pylori* infection that is present in 80-90% of young patients [9,10]. NG may also contribute to the development of diffuse-type gastric carcinoma of the gastric body [4]. Although the mean age of gastric cancer associated with NG is reportedly 34 years, cases in the teens or twenties have also been observed, with 97-100% of patients exhibiting diffuse-type gastric carcinoma [5,11]. NG may be associated with severe pangastritis, which has been strongly linked to a risk of gastric cancer, particularly diffuse-type, in both clinical and basic studies [12-14]. Accordingly, the severity of gastric body inflammation associated with NG has been postulated as one of the most important factors in the carcinogenic pathway of diffuse-type gastric cancer.

Since the relationship between *H. pylori* infection and the development of gastric cancer is well established [12], prompt *H. pylori *detection and eradication may help prevent adverse outcomes [15,16]. Carcinogenesis from NG with active inflammation of the gastric body differs from differentiated carcinogenesis, which is a result of progressive changes from chronic gastritis through atrophy, intestinal metaplasia, and dysplasia [17]. *H. pylori *eradication has been shown to prevent diffuse-type gastric carcinoma associated with NG [11]. However, gastric cancer may still develop after eradication, although rarely at a young age. The youngest reported case of gastric cancer following eradication treatment has been 28 years [18]. To our knowledge, the present patient is now the youngest case to date. Indeed, even if eradication therapy for NG improves gastric mucosal inflammation, the risk of carcinogenesis may exist as early as in the teenage years. This was a young patient with poorly differentiated carcinoma, and so the possibility of hereditary diffuse gastric cancer (HDGC) was considered. Although no genetic analysis was performed, there was no obvious family history of HDGC or other related conditions [19]. Also because HDGC is quite rare in Japan, we ultimately judged this case to be *H. pylori*-related gastric cancer.

Recently, a test-and-treat strategy for *H. pylori* infection targeting mainly junior high school and high school students who frequently present with NG has been implemented in some areas in Japan [9]. However, the follow-up protocol after eradication has not yet been established. In the reported case, advanced diffuse-type gastric cancer was diagnosed only three years after* H. pylori *eradication in an adolescent girl. Although endoscopic examination after eradication therapy is considered beneficial, regular endoscopic follow-up for all young patients treated with eradication is challenging. We have reported that the degree of histological inflammation in the gastric body varies among NG cases; not all NG has the same risk for diffuse-type gastric cancer, and patients with more severe inflammation in the gastric body may be considered high risk for gastric cancer [20]. In the present case, moderate inflammatory cell infiltration in the gastric body was observed in histological evaluations before eradication. Therefore, when performing eradication for NG, it is advisable to estimate the risk of gastric cancer after eradication by assessing histological inflammation in the gastric body by endoscopic biopsy. Further studies are warranted to establish an adequate surveillance program after the eradication for NG, including the optimal examination interval.

## Conclusions

We encountered a 19-year-old patient with advanced gastric cancer occurring three years after* H. pylori *eradication therapy for NG. Assessing histological inflammation in the gastric body by endoscopic biopsy should be done when performing eradication for NG, with careful follow-up for patients with suspicious findings, even in adolescents.
